# TRAF2 regulates the progression of pulmonary fibrosis through β-catenin-Snail signaling pathway

**DOI:** 10.3389/fpubh.2025.1582860

**Published:** 2025-05-14

**Authors:** Zhijie Wan, Jingwen Gu, Wanli Duan, Yuanyuan Chen, Shuya Song, Jingyu Luo, Xide Zhang, Yanyong Yang, Fu Gao, Ying Xu

**Affiliations:** ^1^Department of Radiation Medicine, Faculty of Naval Medicine, Naval Medical University, Shanghai, China; ^2^Shanghai Key Laboratory of Nautical Medicine and Translation of Drugs and Medical Devices, Naval Medical University, Shanghai, China

**Keywords:** TRAF2, pulmonary fibrosis, EMT, β-catenin, Snail

## Abstract

**Introduction:**

Pulmonary fibrosis (PF) is a devastating lung disease characterized by excessive extracellular matrix deposition and impaired pulmonary function, with limited therapeutic options. The pathogenesis of PF involves a complex network of molecular events, including epithelial-mesenchymal transition (EMT), activation of fibroblasts, and dysregulated tissue remodeling. Recent studies have identified TRAF2 (TNF receptor-associated factor 2) as a potential modulator of fibrosis, while its precise mechanism remains unclear.

**Methods:**

We assessed TRAF2 expression and subcellular localization via immunofluorescence and Western blot. TRAF2 knockdown was achieved through siRNA transfection. Protein and mRNA levels of molecules were detected using wb and RT-qPCR. Molecular interactions (TRAF2/β-catenin/Snail) were validated by co-immunoprecipitation assays. HE staining and Masson staining were quantified.

**Results:**

We demonstrate that TRAF2 translocates to the nucleus after fibrosis induction and is positively correlated with disease severity. TRAF2 knockdown significantly reduced collagen deposition and myofibroblast activation, thereby alleviating fibrosis. Furthermore, we investigate the molecular mechanisms by which TRAF2 regulates pulmonary fibrosis, specifically its interaction with β-catenin and Snail, which promotes β-catenin-mediated transcriptional activation and facilitates EMT. These findings offer novel insights into the role of TRAF2 in pulmonary fibrosis, suggesting that TRAF2 may provide a promising therapeutic strategy for this debilitating disease.

**Discussion:**

Our study provides valuable insights into the role of TRAF2 in pulmonary fibrosis, while the precise molecular mechanisms by which TRAF2 interacts with β-catenin and Snail in fibrosis remain unclear. Future studies should aim to explore the mechanisms of TRAF2 in more detail, particularly how it interfaces with fibrotic mediators and cellular processes.

## 1 Introduction

Pulmonary fibrosis (PF) is a progressive and debilitating lung disorder characterized by excessive accumulation of extracellular matrix (ECM) components, leading to irreversible lung damage and impaired gas exchange (1, 2). PF develops through a pathogenic transition from unresolved acute lung inflammation to chronic inflammatory processes (3). The etiology of PF involves multiple contributing factors: recurrent or overwhelming microbial infections; persistent exposure to inorganic particulates including silica and asbestos; systemic autoimmune conditions, particularly rheumatoid arthritis and systemic lupus erythematosus (4). Emerging clinical evidence has further established PF as a significant delayed complication following thoracic radiotherapy, representing one of the most serious late sequelae in cancer treatment (5). Consequently, there is a critical need to identify novel molecular targets for intervention. In recent years, several key molecules have been identified as essential in the development of pulmonary fibrosis. Connective tissue growth factor (CTGF), integrin Dec1, and the receptor tyrosine kinase fibroblast growth factor receptor 1 (FGFR1) promote fibroblast activation and ECM deposition (6, 7). These molecules are known to modulate key fibrotic processes, such as fibroblast proliferation, migration, and collagen synthesis, which contribute to the progression of fibrosis. Importantly, emerging evidence suggests that different pathways often converge on the Wnt/β-catenin signaling axis, a central pathway of fibroblast differentiation, ECM remodeling, and tissue fibrosis (8, 9). Specifically, CTGF has been shown to enhance Wnt signaling through its direct interaction with β-catenin, thereby promoting ECM accumulation and fibrotic progression (10). Similarly, integrin Dec1 can activate TGF-β, which in turn upregulates Wnt/β-catenin signaling, creating a feedforward loop that amplifies fibrosis (11). The Wnt/β-catenin pathway has also been implicated in regulating the expression of Snail, a transcription factor crucial for EMT, which is essential for the progression of fibrosis (12, 13). These complex molecular interactions suggest that Wnt signaling regulates individual fibrotic molecules and orchestrates a broader network of fibrotic responses. However, much remains to be uncovered regarding the precise mechanisms in Wnt signaling pathway.

TRAF2 has emerged as a potential regulator of fibrosis (14, 15). TRAF2 contributes to the activation of hepatic stellate cells and the subsequent overproduction of collagen, driving the progression of fibrotic scarring in liver fibrosis (15, 16). In renal fibrosis, TRAF2 exacerbates tubular epithelial cell injury and interstitial fibrosis, further underscoring its role in fibrotic tissue remodeling. More recent evidence suggested that TRAF2 stimulates fibroblast activation and ECM deposition by modulating pro-inflammatory and pro-fibrotic signaling pathways in pulmonary fibrosis (17). However, the precise molecular mechanism of TRAF2 in pulmonary fibrosis is still unclear. TRAF2 was initially recognized for its involvement in influencing inflammatory responses, cell survival, and apoptosis via its interactions with various signaling pathways, including NF-κB (pro-inflammatory) and JNK (pro-apoptotic) (18, 19). In patients with idiopathic pulmonary fibrosis (IPF), TRAF2 overexpression is correlated with elevated TNF-α levels (20). TNF-α activates Snail transcription via the TRAF2-RIP1-NF-κB signaling axis, thereby promoting tumor migration and EMT (21). Among all signaling pathways regulating EMT, the Wnt/β-catenin pathway plays a pivotal role. Upon nuclear translocation, β-catenin forms a complex with TCF/LEF transcription factors to activate downstream target genes such as Snail, thereby driving the EMT process. Therefore, TRAF2 is a critical regulator of fibrotic pathogenesis by orchestrating the β-catenin/Snail signaling axis. In this study, we investigate the role of TRAF2 in modulating β-catenin and Snail, ultimately influencing fibroblast activation and EMT in pulmonary fibrosis.

## 2 Methods

### 2.1 Cell lines and cell culture

Human pulmonary epithelial cell line A549 was obtained from the American Type Culture Collection (ATCC, USA). Cells were cultured in Dulbecco’s Modified Eagle’s Medium (DMEM; C11965500BT, Gibco, USA), supplemented with 12% fetal bovine serum (FBS; 10099141, Gibco, USA) and 1% penicillin and streptomycin solution (15140122, Thermo Fisher Scientific, USA) in 5% CO₂ at 37°C in a humidified atmosphere. For profibrotic stimulation *in vitro*, cells were treated with 10 ng/mL TGF-β1 (100-21-1MG, Thermo Fisher Scientific, USA) for 48 h after serum starvation, and then processed for downstream experiments. To evaluate Wnt/β-catenin activation in response to TGF-β1 stimulation, cell lysates were collected 6 h after TGF-β1 treatment. For antifibrotic treatment, cells were co-incubated with TGF-β1 and Bufotalin (B20153, Yuanye, Shanghai, China) from the first day and treated continuously for 48 h.

### 2.2 Irradiation conditions

The radiation-induced pulmonary fibrosis model was established using a ^60^Co irradiator (Naval Medical University Irradiation Center, China). Cells were irradiated with 12 Gy at a dose rate of 1 Gy/min. Mice were subjected to local thoracic cavity radiotherapy with a single dose of 25 Gy. The irradiation was conducted under stable temperature and humidity conditions.

### 2.3 RNA sequencing

RNA samples were collected from A549 cells in both the non-irradiation and irradiation groups at 8 hours post-irradiation, followed by RNA extraction (Trizol, Thermo Fisher Scientific). The libraries were sequenced based on the Illumina platform and were performed by Oebiotech (Shanghai, China). *p* values < 0.05 and log2 (fold changes) > 1 were set as thresholds for significant differential expression. We perform hierarchical cluster analysis on differentially expressed genes (DEGs) to prove the expression patterns of genes in different groups and samples. Kyoto Encyclopedia of Genes and Genomes (KEGG) performed a pathway enrichment analysis on DEGs based on the hypergeometric distribution of R, which all genes in TNF signaling pathway and NF-κB signaling pathway, including their overlapping genes in Table 1.

**Table 1:** All genes in TNF signaling pathway and NF-κB signaling pathway, including their overlapping genes.

TNF signaling pathway	NF-κB signaling pathway	Overlap gene
MAPK1, ITCH, RPS6KA5, TRAF3, CHUK, MAP3K7, TAB2, BCL3, PIK3R1, IL1B, MLKL, MMP14, CX3CL1, LTA, TNF, NFKBIA, MAP3K5, IL15, MAP2K6, VEGFC, MAP2K3, ICAM1, MAPK14, JUNB, SELE, PIK3CB, TNFRSF1A, DAB2IP, IRF1, NFKB1, CCL20, CXCL2, CXCL10, MAPK8, TRAF2, TNFAIP3, MAP2K4, CASP7, CXCL1, CXCL5, CXCL3, CREB3L2, DNM1L, SOCS3, PIK3CA, JUN, IL6, TRAF5, AKT3, ATF2, CCL2, PIK3CD, CREB1, CASP8, P3R3URF-PIK3R3	MALT1, BCL2, LYN, TRAF3, CHUK, MAP3K7, TNFRSF13C, CSNK2A1, XIAP, TAB2, PLAU, CCL4, TLR4, IL1B, LTA, TNF, LTB, NFKBIA, EDAR, GADD45A, ICAM1, BCL2A1, DDX58, GADD45B, EDARADD, TNFRSF1A, CARD11, PRKCQ, NFKB1, CXCL2, TRAF2, TNFAIP3, IL1R1, CXCL1, CXCL3, CD14, CXCL8, PIDD1, BCL10, LAT, PLCG2, ATM, TRAF5, ERC1, CARD14	TRAF2, CHUK, MAP3K7, TAB2, IL1B, LTA, TNF, NFKBIA, ICAM1, TNFRSF1A, NFKB1, CXCL2, TRAF3, TNFAIP3, CXCL1, CXCL3, TRAF5

### 2.4 RNA interference assay

The cells were seeded 24 h before transfection to yield a density of 70–80% confluence at the time of transfection. Liposomal cocktails with siRNA (final concentration of 80 nM) were generated using Lipofectamine 3000 (#L3000008, Invitrogen, USA) in Opti-MEM, according to the manufacturer’s recommendations. Fresh culture medium was changed 24 h after transfection. Transfected cells were incubated for 48 h before use. The siRNAs against human TRAF2 were purchased from Hanbio Tech (HH20221201SHQJL, Shanghai, China). The siRNAs are listed in Table 2.

**Table 2:** siRNA sequences used in the article.

Name	Sequence
siRNA-NC	F: UUCUCCGAACGUGUCACGU TT R: ACGUGACACGUUCGGAGAA TT
siRNA-1	F: AGAUGUGUCUGCGUAUCUATT R: UAGAUACGCAGACACAUCUTT
siRNA-2	F: GGACCAAGACAAGAUUGAATT R: UUCAAUCUUGUCUUGGUCCTT
siRNA-3	F: GACGUGACUUCAUCCUCUUTT R: AAGAGGAUGAAGUCACGUCTT

### 2.5 Western blotting analysis

After various treatments, protein was extracted using M-PER Mammalian Protein Extraction Reagent (Thermo Fisher Scientific, USA) with 1% sodium dodecyl sulfate (SDS), protease, and phosphatase inhibitors. The lysates were incubated on ice for 30 min and then boiled in 1 × SDS gel loading buffer for 10 min. Protein concentrations were determined using the BCA Protein Assay Kit (P0010, Beyotime, China). Proteins were separated on 8% or 10% SDS-PAGE gel and transferred to nitrocellulose membranes (Millipore, USA). Membranes were blocked with 5% non-fat milk in TBST at room temperature for 1 h, followed by overnight incubation with primary antibodies at 4°C. On the following day, the membranes were washed three times for 5 min in TBST and incubated with species-specific HRP-conjugated secondary antibodies for 1 h. Next, after three 10-min washes in TBST, membranes were developed using an enhanced chemiluminescence reagent before being exposed to a ChemiDoc Imaging System (6000plus, bltlux, China). Protein bands were quantified using ImageJ software (National Institutes of Health, USA). Antibodies were listed in Table 3.

**Table 3:** The antibody used in this article.

Antibodies	Source	Identifier
TRAF2 (WB)	Cell Signaling Technology	4712
TRAF2 (IF)	Santa Cruz biotechnology	sc-7346
Vimentin	Cell Signaling Technology	5741
E-cadherin	Cell Signaling Technology	3195
α-SMA	Cell Signaling Technology	19245
Col1a1	Abcam	ab260043
β-catenin	Cell Signaling Technology	8480
Snail	Cell Signaling Technology	3879
GAPDH	Cell Signaling Technology	5174
β-Actin	Proteintech	66009-1-ig
HRP conjugated Goat Anti-Rabbit IgG (H + L)	Servicebio	GB23303
HRP conjugated Goat Anti-Mouse IgG (H + L)	Servicebio	GB23301

### 2.6 RNA extraction and quantitative PCR

RNA was extracted using the Total RNA Extraction Kit (RN001, ESScience, China) following the manufacturer’s instructions. RNA concentration and purity were determined using a Nanodrop ND-1000 Spectrophotometer (Thermo Fisher Scientific, USA), and the samples were stored at −80°C. cDNA synthesis was performed with the Script Reverse Transcription Supermix Kit (#RR047A, Takara, Japan) according to the manufacturer’s instructions. Quantitative real-time PCR was carried out using the Power SYBR Green PCR Master Mix (#RR430A, Takara, Japan). For quantification of gene expression, the 2^−ΔΔCT^ method was used, and the data were normalized to an endogenous control (GAPDH). The sequence information for each primer used in gene expression analysis is as follows: *TRAF2* Forward, 5′-TCCCTGGAGTTGCTACAGC-3′; *TRAF2* Reverse, 5′-AGGCGGAGCACAGGTACTT-3′. *GAPDH* Forward: 5′-CAGGAGGCATTGCTGATGAT-3′; *GAPDH* Reverse: 5′-GAAGGCTGGGGCTCATTT-3′.

### 2.7 Co-immunoprecipitation (co-IP)

Cells were lysed in NETN buffer (20 mM Tris–HCl, pH 8.0, 100 mM NaCl, 1 mM EDTA, 0.5% Nonidet P-40) with protease and phosphatase inhibitors. The lysates were centrifuged at 12,000 × *g* for 10 min at 4°C. The supernatant was then incubated with protein G agarose beads and an IgG antibody of the same species as the IP antibody for 10 h at 4°C to reduce non-specific binding. The cleared lysates were then incubated overnight at 4°C with the IP antibody. The beads were thoroughly washed with NETN buffer, boiled in 1 × SDS gel loading buffer for 10 min, and analyzed by Western blotting as described above.

### 2.8 Immunofluorescence (IF)

Cells were planted on glass coverslips and harvested at different time points after irradiation. After washing with PBS, cells were fixed with 4% paraformaldehyde (P1110, Solarbio, China) for 15 min and permeabilised with 0.25% Triton X-100 (T8200, Solarbio, China) for 5 min. For lung tissues, paraffin sections were deparaffinized and rehydrated, followed by antigen retrieval. Cells or sections were blocked with 1% fetal bovine serum (FBS) for 1 to 2 h. After being washed three times with PBS, cells or sections were incubated with primary antibodies at 4°C overnight. After that, cells or sections were washed thoroughly with PBS, then conjugated with Alexa Fluor 488-conjugated anti-mouse antibodies (Invitrogen) and Texas Red-conjugated anti-rabbit antibodies (Vector Laboratories, Burlingame, USA). Images were captured using a fluorescence microscope (ZEISS LSM880, Germany). Clinical samples for lung fibrosis were obtained from the Respiratory Department of the hospital, with ethical approval.

### 2.9 Animal models

Male C57BL/6 mice (average weight approximately 22 g, 6 weeks old) were purchased from the Experimental Animal Center of the Chinese Academy of Sciences (Shanghai, China). The 48 mice were housed in cages with daily bedding changes, maintained at 25 ± 1°C, and had free access to food and water. The lungs of mice received a localized dose of 25 Gy of ionizing radiation. TNIK inhibitor KY05009 (Selleck) was administered via intraperitoneal injection at a working concentration of 50 mg/kg immediately after radiation to the inhibitor treatment group. All mice were allocated into four groups: the control group (*n* = 12), the inhibitor-only group (*n* = 12), the irradiation-only group (*n* = 12), and the combined treatment group (*n* = 12, receiving both the inhibitor and irradiation). For the irradiation-only and combined treatment groups, mice were euthanized at three designated time points—1 week, 4 weeks, and 12 weeks post-irradiation—with four mice sacrificed at each interval to harvest lung tissues. Tissues were processed for hematoxylin and eosin staining and protein extraction, while the remaining tissues were stored at −80°C for future analysis. All animal procedures were conducted according to the ethical guidelines approved by the Animal Care and Use Committee of the Naval Medical University.

### 2.10 Hematoxylin and eosin (HE) staining

Tissues were deparaffinized and rehydrated through a series of graded ethanol solutions. After staining with hematoxylin (C0107, Solarbio, China) for 5 min, sections were differentiated in 1% hydrochloric acid alcohol and then stained with eosin (C0105, Solarbio, Beijing, China) for 2 min. Images were captured using a Zeiss LSM880 light microscope (Germany). The severity of pulmonary fibrosis was assessed using the Ashcroft score, which quantifies fibrosis on a scale of 0 (no fibrosis) to 8 (complete fibrotic destruction).

### 2.11 Masson staining

For Masson’s trichrome staining, lung tissue sections from pulmonary fibrosis models were deparaffinized in xylene, rehydrated through a graded ethanol series, and stained with Weigert’s hematoxylin (5 min) to visualize nuclei. After washing, sections were treated with Biebrich scarlet-acid fuchsin (10 min) to stain cytoplasm and muscle fibers, followed by differentiation in 1% phosphomolybdic acid (10 min) to selectively remove red stain from collagen. Collagen fibers were then counterstained with aniline blue (5 min), dehydrated, and mounted. Fibrotic areas (blue) and cellular components (red and black) were analyzed under light microscopy, with collagen deposition quantified using GraphPad Prism software.

### 2.12 Statistical analysis

Statistical analyses were performed using GraphPad Prism software (version 8.0). Each experiment was independently repeated three times, and the representative data are shown. All the values are presented as mean ± SD of three biologically independent samples. Statistical analyses were performed using a two-way analysis of variance (ANOVA) when comparing at least three groups. The sample size is indicated in the corresponding figure legends. Statistical significance was defined as *p < 0.05 (**p < 0.01; ***p < 0.001; ****p < 0.0001; ns, not significant).

## 3 Results

### 3.1 TRAF2 is upregulated by IR and plays a key role in fibrosis

**Figure 1:**
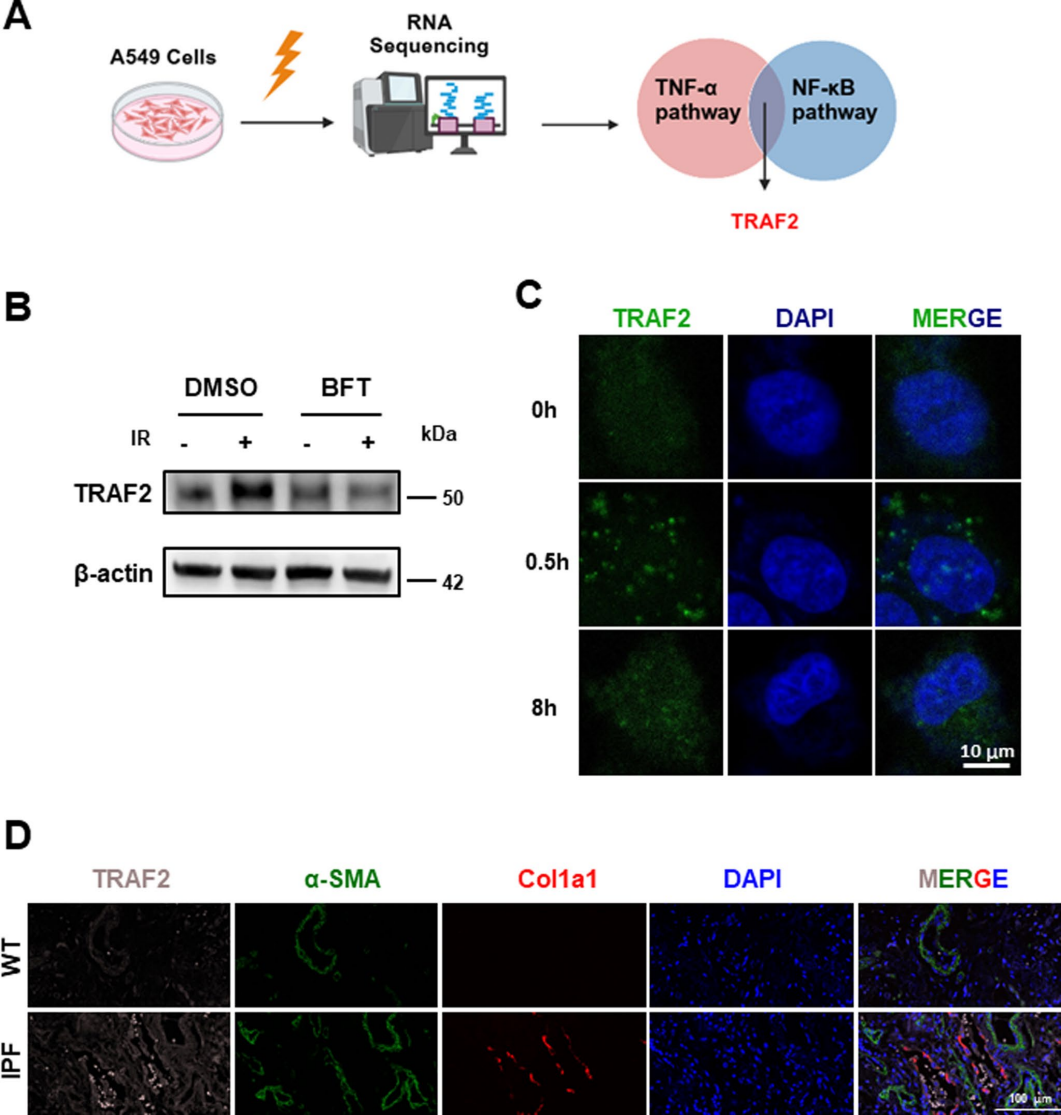
TRAF2 localization and expression in pulmonary fibrosis. **(A)** Schematic representation of the experimental design, showing the upregulation of TRAF2 in A549 cells after IR. **(B)** Western blot analysis of TRAF2 expression in A549 cells after IR and BFT treatment, showing reduced TRAF2 expression following BFT treatment. **(C)** Immunofluorescence showing the nuclear translocation of TRAF2 in A549 cells at different time points (0 h, 0.5 h, and 8 h) after irradiation. The scale bar represents 10 μm. **(D)** Immunofluorescence staining of TRAF2 and α-SMA in lung tissue from control and IPF patients, showing increased TRAF2 expression and localization with α-SMA in IPF tissue. The fluorescent labeling for TRAF2, α-SMA, Col1a1, and DAPI is represented in pink, green, red, and blue, respectively. The scale bar represents 100 μm.

RNA sequencing revealed a significant upregulation of *TRAF2* in response to IR, highlighting its potential involvement in fibrosis-associated signaling pathways, mainly mediated by TNF-α and NF-κB (Figure 1A). Compared with non-irradiation group, 1860 genes were upregulated and 1767 genes were downregulated (Supplementary Figure 1A). Further KEGG pathway analysis identified TRAF2 as a hub gene in the TNF signaling pathway (map04668) and NF-κB signaling pathway (map04064), both critical for inflammation and fibrotic responses (Supplementary Figure 1B; Table 1. Treatment with Bufotalin (BFT), a compound identified for its anti-fibrotic properties (22), effectively inhibited the IR-induced increase in TRAF2 expression, suggesting that TRAF2 may play a crucial role in the progression of fibrosis (Figure 1B). This result indicates that BFT may exert its anti-fibrotic effects, in part, by suppressing TRAF2 expression. Furthermore, to assess the dynamics of TRAF2 localization after IR, immunofluorescence was performed at different time points (0, 0.5, and 8 h) after IR. TRAF2 is predominantly localized in the cytoplasm. However, TRAF2 rapidly translocates to the nucleus at 0.5 h, where it remains elevated until 8 h, indicating its role in nucleus-associated fibrosis (Figure 1C). This nuclear translocation of TRAF2 suggests that it possibly plays a role in activating pro-fibrotic signaling pathways in response to IR. In further validation, we examined TRAF2 expression in lung tissue from patients with IPF and compared it with that of healthy individuals. Immunofluorescence staining revealed that TRAF2 expression is significantly higher in IPF tissue compared to control tissue, with a marked localization of TRAF2 and α-SMA, a well-established marker of myofibroblasts involved in fibrosis (Figure 1D). These findings indicate that TRAF2 may contribute to the activation of myofibroblasts, thus playing a crucial role in the progression of pulmonary fibrosis. Taken together, TRAF2 is a key regulator of the fibrotic response, and its expression is significantly modulated by IR and BFT treatment, as well as in fibrotic lung tissue.

### 3.2 TRAF2 knockdown reduces myofibroblast transition and fibrotic marker expression

**Figure 2:**
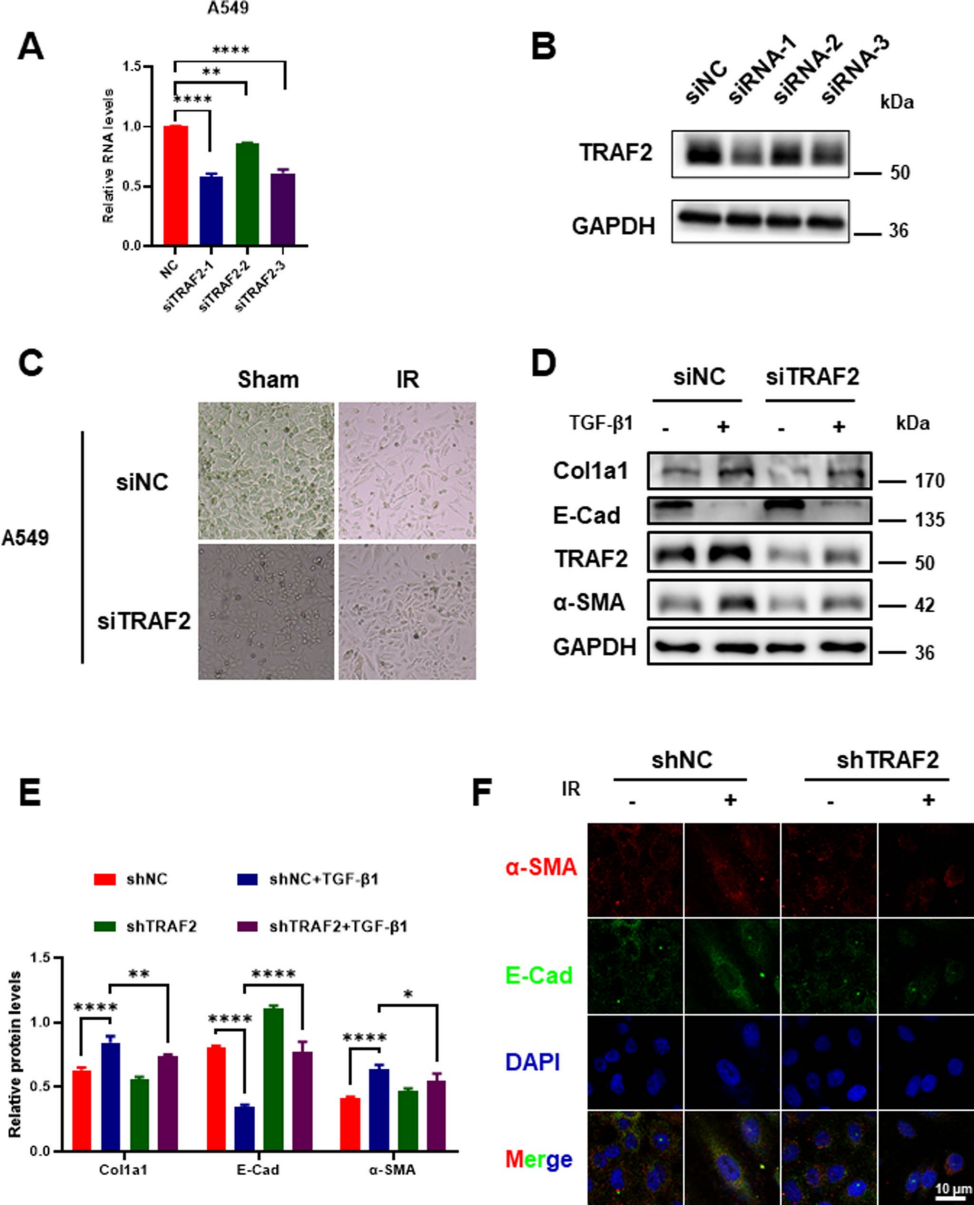
Effect of TRAF2 knockdown on EMT transition in pulmonary fibrosis. **(A)** Relative mRNA expression of *TRAF2* in A549 cells transfected with three distinct siRNA targeting TRAF2 (siRNA-1, siRNA-2, and siRNA-3), showing knockdown effect of TRAF2 expression compared to the negative control group. **(B)** Western blot analysis confirming the knockdown of TRAF2 protein expression in A549 cells transfected with siRNA. **(C)** Morphological changes in A549 cells with TRAF2 knockdown and control (NC) following IR exposure. TRAF2 KD cells exhibited fewer morphological changes after IR exposure compared to the control group. **(D)** The protein expression levels of Col1a1, E-cadherin, TRAF2, and α-SMA in A549 cells with or without TRAF2 knockdown, treated with or without TGF-β1. **(E)** Quantification of relative protein levels for Col1a1, E-cadherin, and α-SMA in A549 cells with TRAF2 knockdown and TGF-β1 treatment. **(F)** Immunofluorescence staining for E-cadherin (red) and α-SMA (green) in A549 cells with or without TRAF2 knockdown and treated with or without IR. DAPI staining marks the nuclei. The scale bar represents 10 μm. **p < 0.01 and ****p < 0.0001 compared to controls. *p < 0.05.

To investigate the role of TRAF2 in the progression of pulmonary fibrosis, we first knocked down TRAF2 in A549 cells using three distinct small interfering RNAs (siRNAs). Among them, the knockdown effect of siRNA-1 on TRAF2 expression was more obvious (Figures 2A,B). In the process of pulmonary fibrosis, the transformation of fibroblasts into myofibroblasts is accompanied by changes in cell morphology and function. Cells exhibited a typical epithelial-like shape under normal conditions, but displayed significant changes, such as cell elongation and loss of cell–cell contacts after IR. However, TRAF2 knockdown cells exhibited fewer morphological changes after IR, suggesting that TRAF2 knockdown may reduce the fibroblastic transition induced by IR (Figure 2C). To further investigate the influence of TRAF2 on the fibrotic response, TRAF2 knockdown cells were treated with TGF-β1, a well-known inducer of fibrosis, which resulted in reduced expression of collagen I (Col1a1) and α-SMA, both key markers of fibrosis (Figure 2D). Knockdown of TRAF2 partially restored the expression of E-cadherin, a protein indicative of epithelial integrity, which was downregulated upon TGF-β1 stimulation (Figure 2D). The protein levels of Col1a1, E-cadherin, and α-SMA were further quantified in Figure 2E, which showed a significant reduction in fibrotic markers and a restoration of epithelial markers in TRAF2 KD cells. Immunofluorescence staining was further performed to confirm the effect of TRAF2 knockdown on E-cadherin and α-SMA localization in A549 cells after IR. α-SMA expression increased after irradiation, while E-cadherin expression decreased, indicative of EMT. However, the magnitude of the change in these two proteins is smaller in TRAF2 KD cells, especially after IR, indicating that TRAF2 knockdown reduces the EMT process (Figure 2F). These findings suggest that TRAF2 is involved in regulating fibrosis-related proteins in response to TGF-β1.

### 3.3 TRAF2 interacts with Snail and β-catenin to enhance fibrotic signaling in pulmonary fibrosis

**Figure 3:**
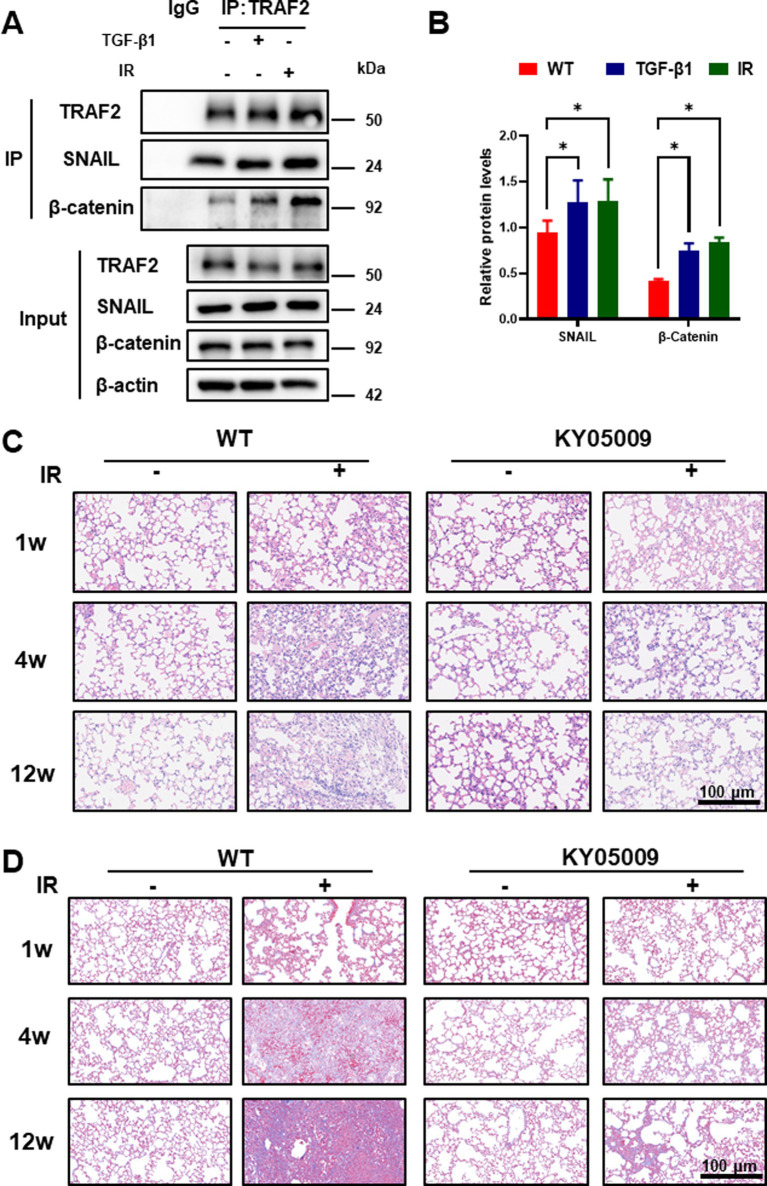
TRAF2 Interacts with Snail and β-Catenin to Enhance Fibrotic Signal in Pulmonary Fibrosis. **(A)** Co-immunoprecipitation (co-IP) assays of TRAF2 interactions with Snail and β-catenin in A549 cells treated with or without TGF-β1 and IR. The input and immunoprecipitated proteins were analyzed by immunoblotting. **(B)** Quantitative analysis of protein expression levels of TRAF2, Snail, and β-catenin in A549 cells treated with TGF-β1 and IR by Western blotting reveals that all three proteins are significantly upregulated after treatment (*p* < 0.05). **(C)** Hematoxylin and eosin (H&E) staining of lung tissue from mice at 1 week, 4 weeks, and 12 weeks after treatment. KY05009 treatment reduced the severity of lung fibrosis, especially at 12 weeks. The scale bar represents 100 μm. Ashcroft scores quantifying the severity of lung fibrosis at 4 weeks and 12 weeks after IR in Supplementary Figure 3C. The IR + KY05009 group displayed significantly lower Ashcroft scores compared to the IR group. *p < 0.05, **p < 0.01, and ****p < 0.0001 compared to controls. **(D)** Masson staining of lung tissue from mice at 1 week, 4 weeks, and 12 weeks after treatment. TNIK inhibition treatment reduced the severity of lung fibrosis, especially at 12 weeks. The scale bar represents 100 μm. Quantification the severity of lung fibrosis at 4 weeks and 12 weeks after IR in Supplementary Figure 2A,B.

We performed co-immunoprecipitation (co-IP) to investigate the interactions between TRAF2 and its downstream effectors in A549 cells treated with TGF-β1 and/or IR. As shown in Figure 3A, TRAF2 was found to interact with both Snail and β-catenin in response to IR exposure. This interaction was significantly enhanced when cells were also treated with TGF-β1, highlighting these two factors in promoting the recruitment of Snail and β-catenin to TRAF2. These results suggest that TRAF2 may facilitate the formation of a complex involving Snail and β-catenin, which could be crucial for the activation of fibrotic signaling pathways. The protein was quantified in Figure 3B, further confirming that treatment with IR and TGF-β1 upregulated the expression levels of TRAF2, Snail, and β-catenin, with the most pronounced increase of Snail and β-catenin observed in treatment group. These findings support the hypothesis that both IR and TGF-β1 promote the stabilization of TRAF2 and its interaction with key transcription factors involved in the development of fibrosis.

### 3.4 Inhibition of TNIK attenuates radiation-induced pulmonary fibrosis *in vivo*

Our findings above revealed that TRAF2 play critical roles in myofibroblast activation and fibrosis. However, the TRAF2-specific inhibitor is unavailable, and the inhibition of TRAF2 often proves insufficient to fully abrogate downstream oncogenic or inflammatory pathways due to compensatory mechanisms and pathway redundancy. TNIK is a downstream effector of TRAF2, playing a critical role in the Wnt/β-catenin and JNK signaling pathways, which regulate cell proliferation, differentiation, and fibrosis (23). Its inhibitors can help overcome the issue of incomplete TRAF2 inhibition. In IPF, TNIK inhibitors (e.g., INS018_055/Rentosertib) target TRAF2-regulated fibrotic pathways, reducing myofibroblast differentiation and extracellular matrix deposition. Clinical trials have demonstrated improved lung function in patients with IPF (24). To evaluate the impact of inhibiting TRAF2 signaling *in vivo*, we utilized the TNIK inhibitor (KY05009) in mice with pulmonary fibrosis induced by irradiation. Histological analysis of lung tissues at different time points (1, 4, and 12 weeks after IR) with HE and Masson staining is shown in Figures 3C,D. No significant lung damage was observed in NC group, whereas the lung tissues showed progressive fibrosis with increased collagen deposition over time in the IR group. Treatment with KY05009 significantly reduced the extent of fibrosis at all time points, particularly at 12 weeks, as evidenced by less severe pathological changes in the IR + KY05009 group. These results suggest that inhibiting the TRAF2-TNIK pathway can attenuate the progression of pulmonary fibrosis. Quantification of lung fibrosis was performed using the Ashcroft scoring system, as shown in Supplementary Figure 2A,B. The Ashcroft scores at 4 weeks and 12 weeks after IR were significantly lower in the IR + KY05009 group compared to the IR group, with the difference being most significant at 12 weeks. These results indicate that the inhibition of TRAF2 signaling via TNIK suppresses the progression of lung fibrosis induced by IR.

### 3.5 Inhibition of TRAF2 signaling by TNIK inhibitor mitigates collagen deposition and fibrosis

**Figure 4:**
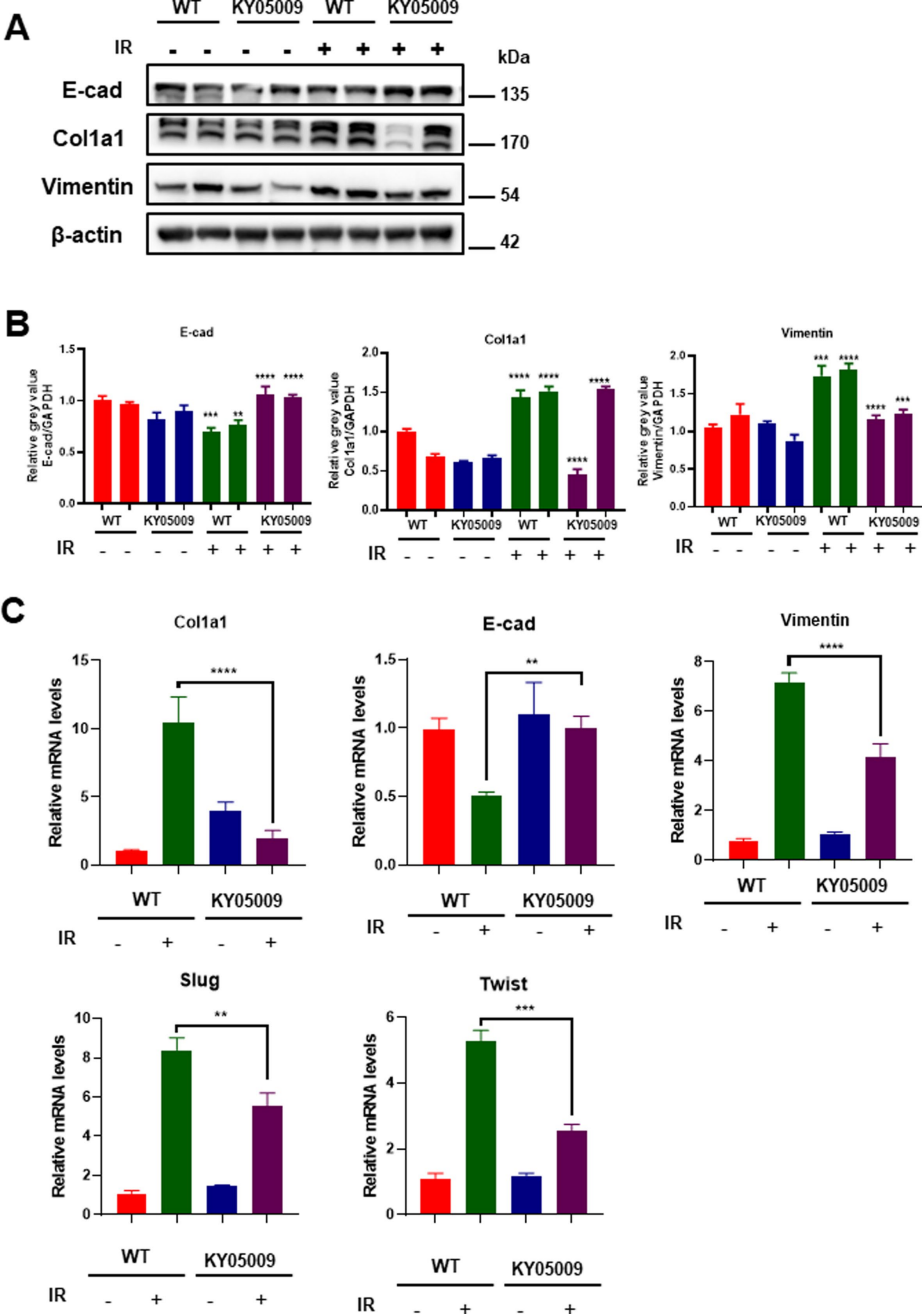
TNIK-mediated TRAF2 inhibition reduces fibrosis and EMT in pulmonary tissue. **(A)** Western blot analysis of E-cadherin, Col1a1, and vimentin expression in lung tissue treated with KY05009, IR, and IR + KY05009, with actin as a loading control. **(B)** Quantification of protein levels for vimentin, Col1a1, and E-cadherin in the experimental groups revealed significant differences between the IR and IR + KY05009 groups (*p* < 0.05, *p* < 0.01). **(C)** Relative mRNA expression of *Col1a1*, *vimentin*, *E-cadherin*, *Slug, and Twist* in the WT, KY05009, IR, and IR + KY05009 groups, showing a significant reduction in fibrosis markers and restoration of E-cadherin with TNIK inhibition treatment. **p < 0.01, ***p < 0.001, and ****p < 0.0001 compared to controls.

Furthermore, we assess the expression of key proteins involved in EMT and fibrosis, including E-cadherin, Col1a1, and vimentin, in lung tissue through Western blot analysis. As shown in Figure 4A, significant upregulation of Col1a1 and vimentin, along with a decrease in E-cadherin, was observed in tissue treated with IR, indicating the induction of fibrosis and EMT. However, the combination of IR and TNIK inhibiting treatment (IR + KY05009) significantly reduced the expression of fibrotic markers (Col1a1 and vimentin) and restored E-cadherin expression, suggesting that TNIK-mediated inhibition of TRAF2 mitigates the fibrotic response. Quantification of protein levels in Figure 4B confirmed these results. Vimentin expression was significantly reduced in the IR + KY05009 group compared to the IR group, and the expression of Col1a1 showed a similar trend with considerably lower levels in the IR + KY05009 group compared to the IR group. Moreover, E-cadherin expression was restored in the IR + KY05009 group, reaching levels comparable to those in the WT group, highlighting the protective effect of TRAF2 inhibition. Relative mRNA expression levels of *Col1a1*, *vimentin*, *E-cadherin, Slug and Twist* were quantified across all experimental groups (Figure 4C). The results show that the IR group exhibited a significant increase in Col1a1 and vimentin expression, while E-cadherin was downregulated. However, treatment with TNIK inhibitor (in the IR + KY05009 group) significantly reduced Col1a1 and vimentin expression, while restoring E-cadherin expression to levels comparable to those in the control. These findings suggest that inhibiting TRAF2 with TNIK effectively reduces fibrosis and EMT in response to IR.

## 4 Discussion

**Figure 5:**
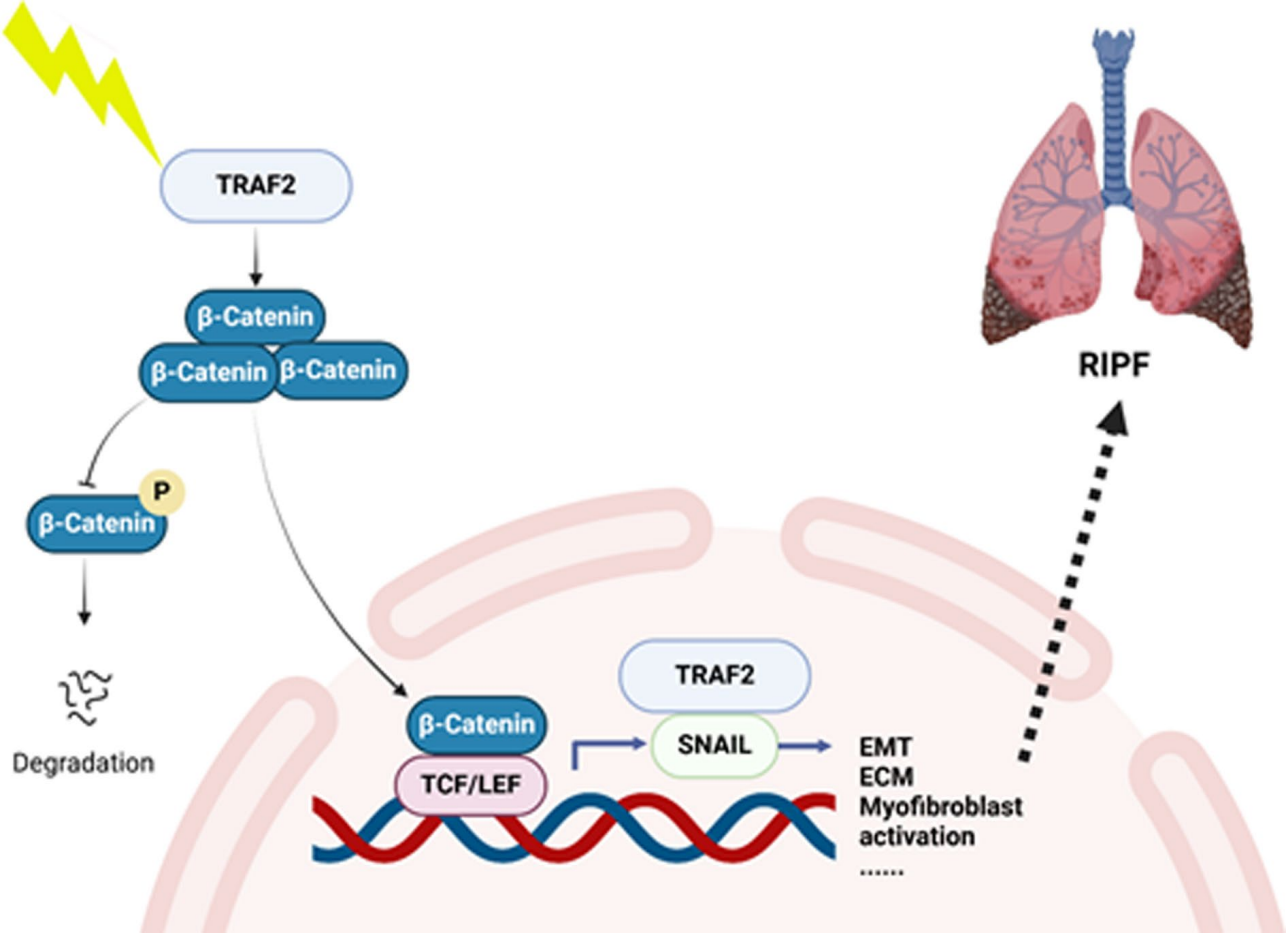
A hypothetical model of TRAF2 involved in pulmonary fibrosis. In this study, we identified TRAF2 as a crucial regulator in the progression of pulmonary fibrosis. We proposed a hypothetical model that TRAF2 interacts with β-catenin and Snail in nucleus to promote EMT and activate fibroblast during fibrosis.

Pulmonary fibrosis is a progressive interstitial lung disease characterized by the excessive deposition of extracellular matrix components in the lung parenchyma, resulting in architectural distortion, impaired gas exchange, and ultimately, respiratory failure. It is commonly associated with various etiologies, including environmental exposures, autoimmune diseases, and genetic factors. The pathogenesis of PF involves the dysregulation of multiple molecular pathways, including inflammation, EMT transition, and fibroblast activation, with key signaling molecules such as TGF-β, TNF-α, and NF-κB playing critical roles in disease progression. In this study, we identified TRAF2 as a crucial regulator in the progression of pulmonary fibrosis. We proposed a model in which TRAF2 interacts with β-catenin and Snail, two pivotal molecules involved in the EMT and fibroblast activation during fibrosis (Figure 5). We observed that TNIK inhibitors target TRAF2-regulated fibrotic pathways, thereby alleviating fibrotic changes and significantly reducing collagen deposition, myofibroblast differentiation, and EMT. These findings suggest that TRAF2 plays a crucial role in fibroblast activation and serves as a critical modulator of the Wnt/β-catenin signaling pathway in pulmonary fibrosis. To the best of our knowledge, the study directly links TRAF2 to the modulation of β-catenin-mediated transcriptional activation and Snail-driven EMT in the context of PF.

Previous studies have highlighted the critical role of the Wnt/β-catenin signaling pathway in the development of pulmonary fibrosis (25). β-catenin activation has been shown to enhance fibroblast proliferation, differentiation, and ECM deposition, contributing to the progression of fibrosis (26). Snail, another key player in fibrosis, is involved in the induction of EMT, a process through which epithelial cells acquire fibroblast-like properties, facilitating tissue remodeling and fibrosis (27). Many studies have explored the involvement of these pathways in fibrosis, while the role of TRAF2 in regulating these pathways has remained underexplored. Our study elucidated how TRAF2 functions as a modulator of both the Wnt/β-catenin signaling axis and Snail-mediated EMT. We demonstrate that the interaction of TRAF2 with β-catenin and Snail is essential for the progression of pulmonary fibrosis, thereby distinguishing our findings from those that focus solely on either β-catenin or Snail individually. TRAF2 orchestrates pulmonary fibrosis through a sophisticated signaling network that converges on multiple pathogenic pathways (28). As a central adaptor protein, TRAF2 activates canonical NF-κB signaling by recruiting the IKK complex to TNF receptors through the K63-linked polyubiquitination of RIP1 (29), leading to the nuclear translocation of p50/p65 heterodimers that transactivate pro-fibrotic cytokines (TNF-α, IL-6) and EMT regulators (Twist1) (30). Simultaneously, TRAF2 mediates MAPK activation through the formation of stress-responsive ASK1-TRAF2 complexes (31), resulting in the JNK-dependent phosphorylation of c-Jun, which enhances AP-1 activity, and p38-mediated stabilization of α-SMA mRNA (32). Furthermore, TRAF2’s physical interaction with TGF-β receptors amplifies Smad2/3 phosphorylation (33), as well as its coordination with Wnt/β-catenin signaling through Disheveled binding (34). The fibrogenic potential of this network is modulated by negative regulators including CYLD-mediated deubiquitination (35). This multivalent signaling architecture explains the limited efficacy of single-pathway inhibitors in clinical trials and underscores the therapeutic potential of targeted TRAF2 degradation strategies.

Currently, antifibrotic therapies, such as pirfenidone and nintedanib, show limited effectiveness and only slow disease progression, without reversing fibrosis (36). Therefore, there is an urgent need for novel therapeutic strategies targeting key molecular regulators in fibrosis. TRAF2, as a crucial mediator in the fibrotic response, presents a promising target for intervention. The identification of TRAF2 as a critical regulator in pulmonary fibrosis is particularly significant given the increasing prevalence of fibrotic lung diseases, including idiopathic pulmonary fibrosis (IPF) and radiation-induced pulmonary fibrosis. Our study provides valuable insights into the role of TRAF2 in pulmonary fibrosis, while the precise molecular mechanisms by which TRAF2 interacts with β-catenin and Snail in fibrosis remain unclear. Future studies should aim to explore the mechanisms of TRAF2 in more detail, particularly how it interfaces with other fibrotic mediators and cellular processes. For instance, investigating how TRAF2 affects fibroblast-to-myofibroblast differentiation and ECM remodeling would provide deeper insights into its role in pulmonary fibrosis. Developing TRAF2 inhibitors and evaluating their potential efficacy and safety in clinical trials will be crucial for translating these findings into novel therapeutic strategies. Moreover, combining TRAF2 inhibition with other existing treatments, such as pirfenidone or nintedanib, may provide synergistic effects in managing pulmonary fibrosis. Investigating these combinations in both preclinical and clinical settings will help determine the most effective therapeutic approaches for patients with fibrotic lung diseases. Furthermore, expanding the research to include chronic fibrotic models will provide more comprehensive insights into the potential of targeting TRAF2 as a therapeutic strategy in pulmonary fibrosis.

Research on pulmonary fibrosis has garnered significant attention in recent years, particularly in elucidating its molecular mechanisms and identifying new therapeutic targets. A significant amount of focus has been placed on the role of key signaling pathways, such as TGF-β, Wnt/β-catenin, and NF-κB, in driving the development of fibrosis (5). Studies have highlighted the role of β-catenin in promoting fibroblast activation and ECM accumulation, which are key features of pulmonary fibrosis (26). While significant progress has been made in understanding the role of TGF-β and other cytokines in pulmonary fibrosis, the role of TRAF2 in this context remains largely unexplored. Therefore, our study focuses on identifying new molecular targets in the treatment of pulmonary fibrosis, providing an important foundation for future investigations into TRAF2-targeted therapies.

## 5 Conclusion

In conclusion, our study provides novel insights into the role of TRAF2 in pulmonary fibrosis, highlighting its interaction with β-catenin and Snail in regulating key fibrotic pathways. TRAF2 inhibition emerges as a promising therapeutic strategy, with the potential to alter the course of pulmonary fibrosis and improve patient prognosis. While several challenges remain to be addressed, particularly regarding the long-term effects and translation into clinical practice, this research lays the groundwork for future studies targeting TRAF2 in pulmonary fibrosis and other fibrotic diseases.

## Data Availability

The original contributions presented in the study are included in the article/Supplementary material, further inquiries can be directed to the corresponding authors.
