# Supplementary material for: TRAF2 regulates the progression of pulmonary fibrosis through β-catenin-Snail signaling pathway
Source: Front Public Health. 2025 May 14;13:1582860. doi: 10.3389/fpubh.2025.1582860 (PMC12116375; doi:10.3389/fpubh.2025.1582860)
Supplement: Supplementary file 1 [file Presentation_1.pdf]

Supplementary Figure

Figure S1

A B

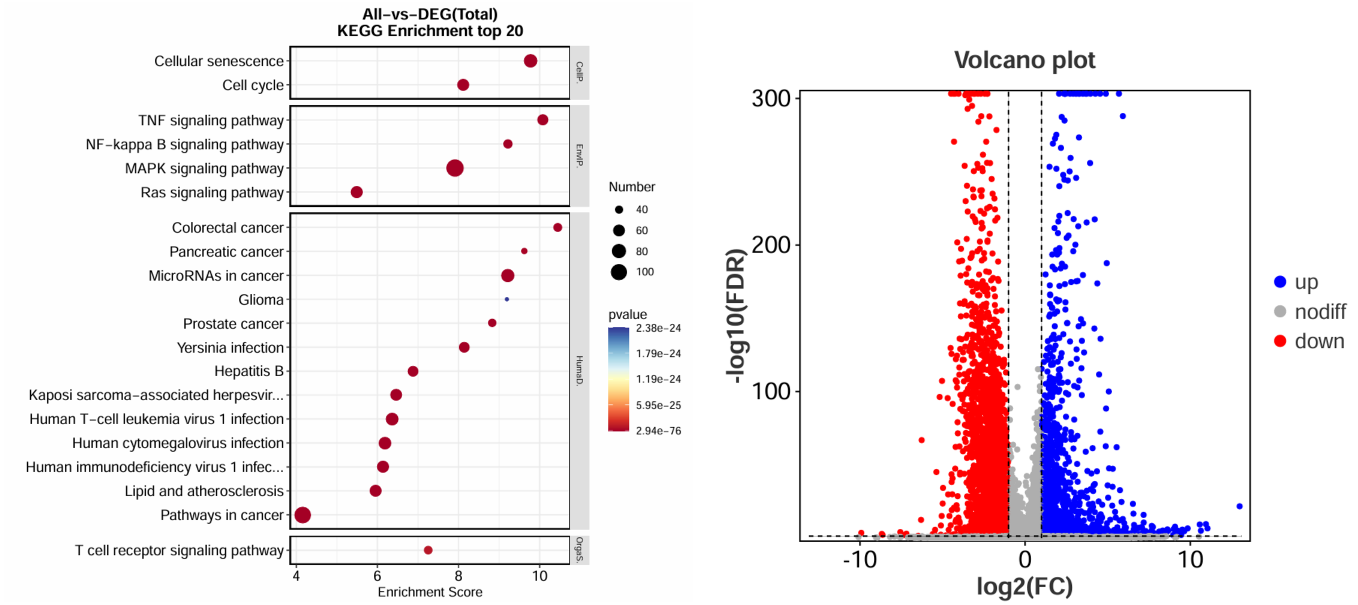

Figure S1

(A) Volcano plot analysis of differentially expressed genes ($p < 0.05$, |log2FC|≥1) in irradiated versus unirradiated cells. A total of 1,767 genes were downregulated, and 1,860 genes were upregulated. **(B)** Top 20 KEGG pathway enrichment analysis of differentially expressed genes.

Supplementary Figure
Figure S2

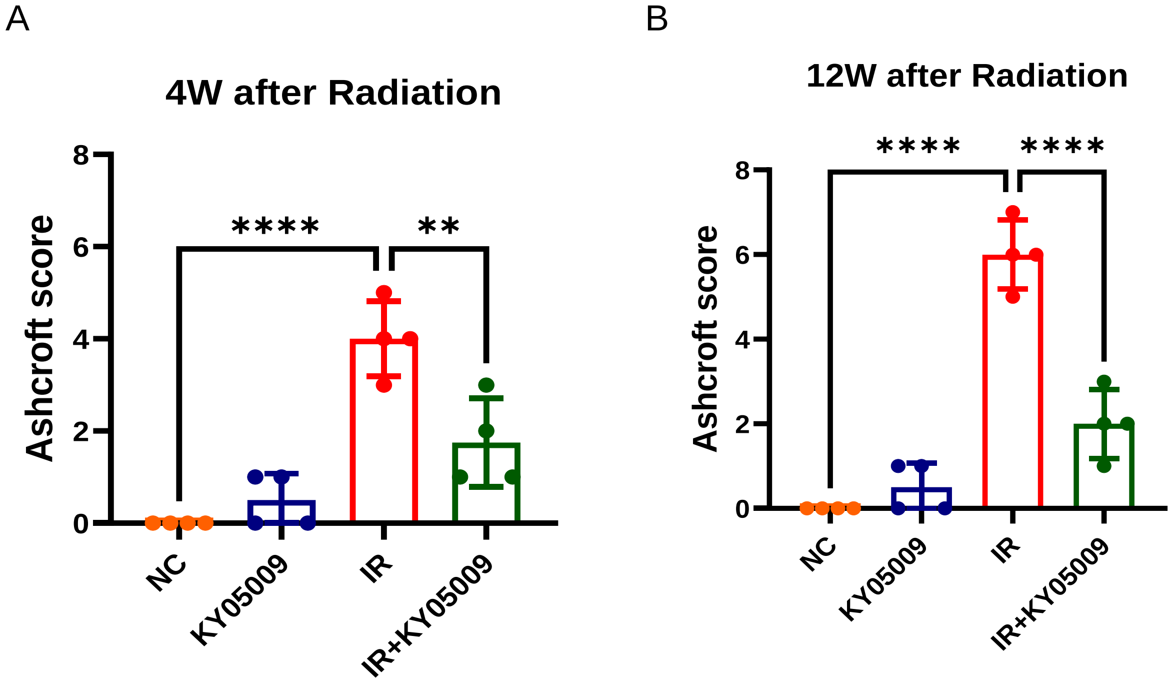

Figure S2
(A) Ashcroft scoring analysis of fibrosis in lung tissue 4 weeks after irradiation. **(B)** Ashcroft scoring analysis of fibrosis in lung tissue 12 weeks after irradiation.

Figure1B

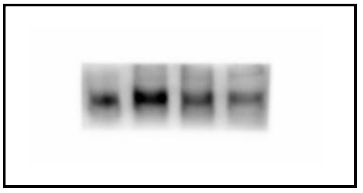

TRAF2

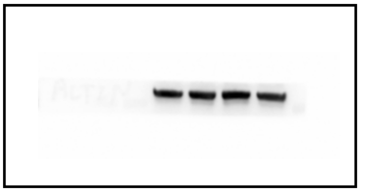

β-actin

Figure2B siTRAF2

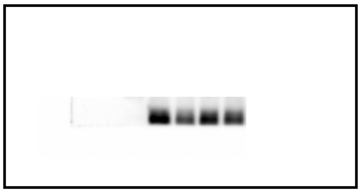

TRAF2

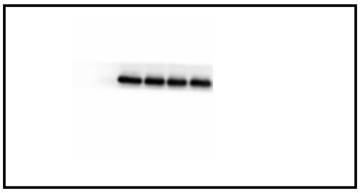

GAPDH

Figure2D EMT

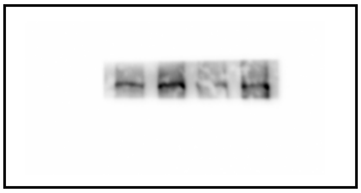

Col1a1

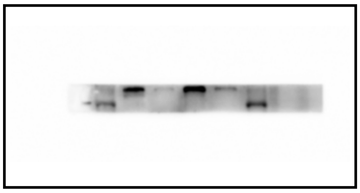

E-Cad

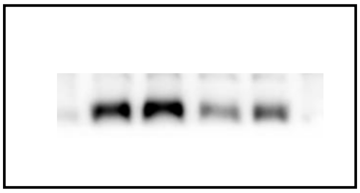

TRAF2

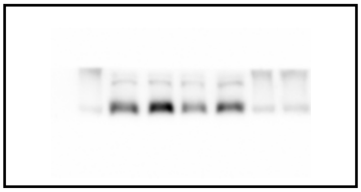

α-SMA

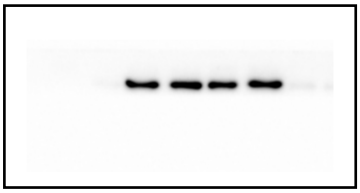

GAPDH

Figure3A IP

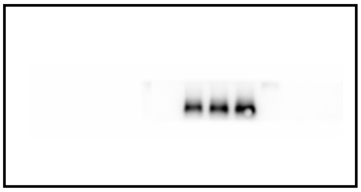

TRAF2 IB

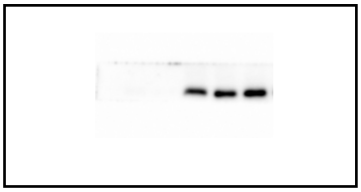

SNAIL IB

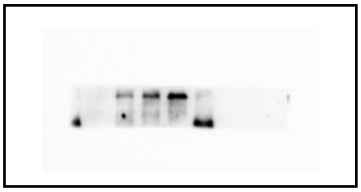

β-catenin IB

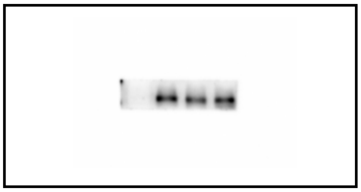

TRAF2 input

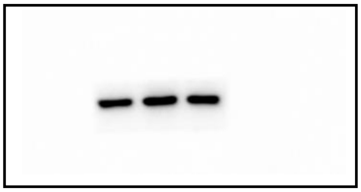

SNAIL input

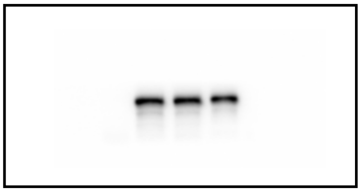

β-catenin input

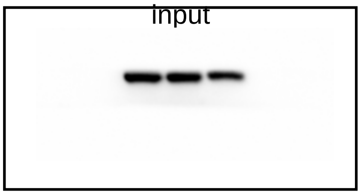

β-actin input

Figure4A

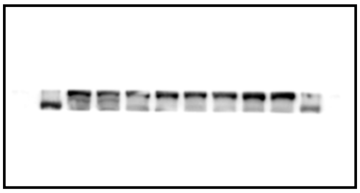

E-Cad

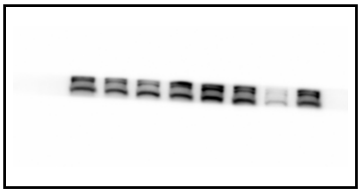

Col1a1

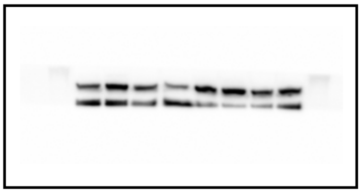

Vimentin

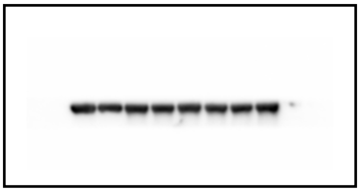

β-actin
